# Cardiac Involvement in Eosinophilic Granulomatosis with Polyangiitis: A Meta-Analysis of 62 Case Reports

**Published:** 2020-01

**Authors:** Mojdeh Pakbaz, Marziyeh Pakbaz

**Affiliations:** 1 School of Medicine, Shahid Beheshti University of Medical Sciences, Tehran, Iran.; 2 Department of Cardiovascular Diseases, Hazrat‐e Rasool General Hospital, Iran University of Medical Sciences, Tehran, Iran.

**Keywords:** *Churg-Strauss syndrome*, *Anti-neutrophil cytoplasmic antibody-associated vasculitis*, *Cardiovascular diseases*

## Abstract

**Background:** Eosinophilic granulomatosis with polyangiitis (EGPA) is a rare multi-systemic vasculitis, with cardiac involvement being one of its most serious manifestations. We aimed to systematically review and analyze the limited case reports of EGPA with cardiac involvement.

**Methods:** Based on the Preferred Reporting Items for Systematic Reviews and Meta-Analyses guidelines, we performed a systematic literature search for the case reports of EGPA with cardiac involvement in the MEDLINE database from 2011 until 2018. For each case, clinical data including sex, age, clinical presentation, electrocardiographic and cardiac imaging findings, the type of cardiac involvement, the available laboratory data (cardiac biomarkers, white blood cell count, eosinophilic count, erythrocyte sedimentation rate, C-reactive protein, and antineutrophil cytoplasmic antibody positivity), therapeutic regimen, and the outcome of the patients were collected and analyzed.

**Results:** A total number of 62 cases were included. The mean age was 48.29±15.60 years, and 51.6% were male. All the cases were in the active disease state. Cardiac symptoms, electrocardiographic abnormalities, abnormal biomarkers, and abnormal echocardiography were detected in 82.3%, 68.5%, 77.4%, and 96.8%, respectively. Cardiac magnetic resonance was done in 46.8% of the patients, and it was abnormal in all. The most common abnormal findings in echocardiography were systolic left ventricular dysfunction (83.9%) and pericardial effusion (37.1%). The most common type of clinical presentation was clinical heart failure (51.6%). Only 6.5% of the patients presented with tamponade. The overall prognosis was good.

**Conclusion:** Any part of the heart could be involved by EGPA. The results emphasize the necessity of in-depth cardiac evaluation in these patients.

## Introduction

Eosinophilic granulomatosis with polyangiitis (EGPA), historically known as Churg–Strauss syndrome, is a rare multi-systemic disease characterized by asthma, the necrotizing vasculitis of small vessels with extravascular granuloma, and marked eosinophilia.^1^ Traditionally, EGPA has been described to evolve through 3 phases^2^: 1) the prodromal phase: Bronchial asthma is the main manifestation of this phase presenting in 96.0–100.0% of patients (The majority of patients in this phase also suffer from otolaryngological involvements.); 2) the eosinophilic phase: This phase is characterized by peripheral eosinophilia with the eosinophilic infiltration of specific organs including the lung, heart, and gastrointestinal (GI) tract; and 3) the vasculitic phase: The cardinal manifestation of this phase is peripheral neuropathy occurring in 70.0% of patients.^3^ Other features of this phase are skin lesions, kidney involvement, and the central nervous system (CNS) manifestations presenting in 67.0%, 25.0%, and 8.0% of patients, respectively.^2^^, ^^4^^, ^^5^

The prognosis and treatment of an individual patient with EGPA depend on the type and severity of organ involvement. The five-factor score (FFS) has been proposed to predict the prognosis of patients with EGPA and it consists of the following elements: elevated serum creatinine levels, proteinuria, the GI tract involvement, cardiomyopathy, and the CNS involvement.^6^

A score of 1 is allocated for each component. The prognosis of patients with an FFS ≥1 is worse, and these patients should be treated with a combination of glucocorticoids and immunosuppressants,^7^ whereas glucocorticoid therapy alone is recommended in those with an FFS=0.8.^8^

EGPA is one of the most common of the systemic vasculitides to affect the heart.^9^ The reported frequency of cardiac involvement varies between 16.0% and 29.0% in different studies.^10^ Cardiac involvement is of great clinical importance because it is the major cause of morbidity and mortality in these patients in spite of the overall good prognosis of EGPA.^11^ Fifty-percent of deaths in patients suffering from EGPA are related to cardiac diseases.^9^ A prompt diagnosis of cardiac involvement and the commencement of appropriate treatment may improve the overall outcome of these patients. 

With regard to the rarity of this clinical entity and the importance of cardiac involvement in these patients, we decided to systematically review the case reports of EGPA with cardiac involvement. Our information was collected from EGPA case reports in the medical literature with documented cardiac involvement from 2011 to 2018.

## Methods

**Figure 1:**
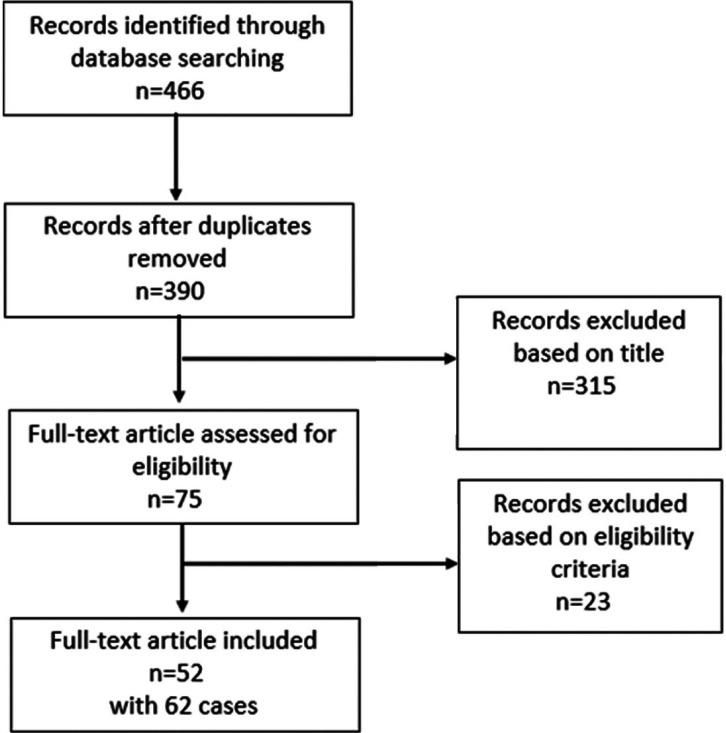
Flow chart describing the search strategy and study selection

Based on the Preferred Reporting Items for Systematic Reviews and Meta-Analyses (PRISMA) guidelines,^12^ case reports of EGPA with cardiac involvement were searched in the MEDLINE database using the following MeSH terms: “Eosinophilic granulomatosis with polyangiitis” OR “Churg–Strauss syndrome” AND “case report(s)” AND “cardiac involvement”. The preliminary search resulted in 446 records, which were screened for duplicated items and then assessed for eligibility for inclusion (Figure 1). The eligibility criteria were as follows: 1) patients > 15 years of age, 2) case reports/series with available full-texts, 3) case reports/series published from January 2011 to May 2018, 4) case reports/series published in English, and 5) case reports/series with documented cardiac involvement caused by EGPA. The data on the enrolled case reports were collected for further analysis. The collected clinical data were entered into a Microsoft Excel database. The data were comprised of the first author, the year of publication, sex, age, clinical presentation, history of asthma, possible extracardiac involvement (lung, skin, kidney, GI, ear-nose-throat, and the nervous system), electrocardiographic findings (arrhythmia, ST-T changes, and conduction disorders), cardiac imaging findings (including echocardiography, angiography, and cardiac magnetic resonance [CMR]), the results of endomyocardial biopsy or extracardiac biopsy, the left ventricular ejection fraction (LVEF) at the time of presentation and during the follow-up, the type of cardiac involvement (pericardial effusion, pericarditis, cardiomyopathy, myocarditis, valvular abnormalities, intracardiac thrombus formation, and evidence in favor of coronary arteritis), the available laboratory data (cardiac troponin T/I, b-type natriuretic peptide [BNP], N-terminal pro b-type natriuretic peptide [NT-pro BNP], the white blood cell [WBC] count, the eosinophilic count, the percentage of eosinophilia, the erythrocyte sedimentation rate, C-reactive protein, and antineutrophil cytoplasmic antibody [ANCA] positivity), therapeutic regimen, and the outcome of the patients.

The continuous variables were expressed as the mean±the standard deviation (SD), and numbers and percentages were used for the categorical variables. The demographic data including age and sex were analyzed. The prevalence of cardiac involvement and other organ diseases was calculated. Organ involvement was calculated as the percentage of affected individuals. Paraclinical data including ECG findings, cardiac imaging findings, biomarkers, ANCA positivity, the WBC count, and the eosinophil count were assessed and analyzed. The types of treatment were defined and categorized, and the number and percentage of the patients in each treatment category were calculated. The overall prognosis (recovery, relapse, and mortality) was analyzed.

## Results

Our systematic search of the case reports of patients suffering from EGPA with cardiac involvement yielded 52 full-text articles featuring 62 cases.^4^^, ^^13^^-^^63^ All the patients fulfilled the American College of Rheumatology criteria of EGPA.^64^ The mean age was 48.29±15.60 years (range=16–77 y). Thirty-two (51.6%) patients were male. The baseline characteristics of our study cohort are summarized in Table 1. The active disease state was evident in all the patients based on the average value of C-reactive protein, the WBC count, and the eosinophil count. 

**Table 1:** Baseline characteristics of the EPGA cohort*

Variables	
Male	32 (51.6%)
Age (y)	48.29±15.60
Asthma	58 (93.5%)
Blood eosinophilia >10%	44 (97.8%)
Mononeuropathy or polyneuropathy	22 (35.5%)
Non-fixed pulmonary infiltrates	35 (56.4%)
Paranasal sinus abnormalities	38 (61.3%)
Available histology with tissue eosinophilia	35 (56.4%)
Mean 5 factor score	1.06±0.83
Erythrocyte sedimentation rate (mm first hour)	49.25±19.21
C-reactive protein (mg/dL)	17.65±32.88
Leukocyte count (x1000/ µL)	18.66±8.51
Eosinophil count (x1000/ µL)	10.11±10.02
Eosinophils (% of leukocyte count)	44.29±21.10
ANCA+	9 (17.0%)
Rise of cardiac biomarkers	48 (77.4%)

EGPA, Eosinophilic granulomatosis with polyangiitis; ANCA, Antineutrophil cytoplasmic antibody

* Data are presented as n (%) or mean±SD.

Cardiac assessment revealed cardiac symptoms in 51 (82.3%) patients, ECG abnormalities in 37 (68.5%), abnormal cardiac biomarkers in 48 (77.4%), and abnormal echocardiography in 60 (96.8%). CMR was done for 29 (46.8%) patients, and it was abnormal in all; thus, it is probably the most sensitive method for detecting cardiac involvement in patients with EGPA. Abnormal echocardiographic findings in our study population consisted of abnormal LV systolic function in 52 (83.9%), more-than-mild diastolic dysfunction in 2 (3.2%), regional wall motion abnormalities in 6 (9.7%), ventricular hypertrophy in 8 (12.9%), valvular regurgitation in 8 (12.9%), pulmonary hypertension in 4 (6.5%), pericardial effusion in 23 (37.1%), and intracardiac thrombi in 14 (22.6%) cases. Echocardiography was completely normal in only 2 cases.^30^^, ^^39^ In both patients, the cardiac troponin level had risen. Of 20 patients presenting with chest pain, 11 cases underwent coronary angiography. Significant coronary involvement was detected only in 4 (36.4%) patients. Normal coronary arteries or mild coronary lesions were detected in 63.6%. Sixteen patients underwent endomyocardial biopsy. A predominant neutrophilic infiltrate was detected in 1 case, and a lymphocytic infiltrate was detected in another 2 cases. In all the remaining 13 cases, eosinophilic myocarditis was reported. In some patients with proven myocarditis based on biopsy and/or CMR, the LV function was preserved.

Patients with EGPA present with a highly variable clinical picture; we, accordingly, classified our study population as follows: 1) Patients who presented with subclinical cardiac involvement. These patients presented with mainly non-cardiac symptoms including constitutional symptoms (lethargy, fatigue, and fever), respiratory presentations (cough and hemoptysis), neurological symptoms (neuropathy), the GI symptoms, and skin lesions. Cardiac involvement in these patients was discovered based on LV dysfunction detected in cardiac imaging (echocardiography and/or CMR), ECG abnormalities, and abnormal cardiac biomarkers. Of the total study population, 11 (17.7%) cases were placed in this group. 2) Patients who presented with heart failure syndrome. This was the most common type of presentation and was observed in 32 (51.6%) patients. A subgroup of these patients (7 [11.3%] cases) had more hemodynamic impairment at the time of presentation and was diagnosed with cardiogenic shock. 3) Patients who presented with chest pain. This was a relatively common presentation of the patients with EGPA and was evident in 20 (32.3%) cases. The potential causes of chest pain were coronary vasculitis resulting in coronary spasm and/or intracoronary thrombosis, pericarditis or myocarditis, and occasionally coincidental atherosclerosis. 4) Patients who presented with cardiac tamponade. Variable amounts of pericardial effusion were a relatively common finding in our study population (37.1% of the cases), but cardiac tamponade as the initial presentation was reported in only 4 (6.5%) cases. 5) Patients who presented with palpitation. Palpitation was the main presenting symptom in only 2 cases, which was due to atrial arrhythmia (atrial fibrillation with rapid ventricular response) in 1 case^30^ and ventricular arrhythmia (polymorphic premature ventricular beats) in the other case.^61^

In our study population of patients suffering from EGPA with cardiac involvement, the most common extracardiac organs involved were the lungs (excluding asthma) and the ear-nose-throat system (Table 2). The peripheral nervous system was also commonly involved, usually manifesting in the form of mononeuritis multiplex. The skin was also affected presenting with nodules or rash. In a case reported by Tsugu et al.,^47^ a positive skin biopsy without an apparent skin lesion aided the diagnostic approach. The CNS involvement, as well as the kidney and GI involvement, was not a common presentation in our study population. The CNS vasculitis presented with ischemic brain lesions,^15^^, ^^39^^, ^^55^ subarachnoid hemorrhage,^54^ seizure,^36^ or rarely with oculomotor nerve palsy.^16^ In a case reported by Bujak et al.,^45^ the patient presented with the CNS symptoms due to brain ischemia but it was actually due to embolization from an LV thrombus. Renal involvement was not common in our study population with regard to ANCA positivity (only 9 cases). Two of these ANCA-positive EGPA cases presented with significant proteinuria and a rise in creatinine, suggesting acute glomerulonephritis.

The pericardium is one of the targets for cardiac involvement among patients with EGPA. Pericardial involvement maybe manifested as variable amounts of pericardial effusion or pericarditis. The patients of our study population most commonly had mild amounts of effusion. The presence of effusion was not essentially accompanied by the symptoms and signs of pericarditis. As was previously mentioned, tamponade was an uncommon presentation in our study population. In an interesting case of EGPA introduced by Yano et al.,^38^ the patient presented with tamponade, mononeuritis multiplex, skin lesions, and diarrhea, and pericarditis was confirmed by the observation of late gadolinium enhancement in the pericardium in CMR. A unique case by Suganuma et al.^16^ showed 2 rare manifestations of EGPA: tamponade and subsequently oculomotor nerve palsy. The patient presented with double vision, which developed 18 days after an episode of cardiac tamponade. 

**Table 2:** Eosinophilic granulomatosis with polyangiitis (EGPA) manifestations

Manifestation	n =62	Manifestation	n =62
Ear-nose-throat	38 (61.3)	Heart	
Sinusitis	30 (48.4)	Subclinical involvement	11 (17.7)
Nasal polyps	15 (24.2)	Clinical heart failure	32 (51.6)
Rhinitis	10 (16.1)	Cardiogenic shock	7 (11.3)
Epistaxis	1 (1.6)	Chest pain	20 (32.3)
Available nasal biopsy positive for tissue eosinophilia	5 (8.1)	Arrhythmia	17 (27.4)
Peripheral nervous system	23 (37.1)	Pericardial effusion	23 (37.1)
Mononeuritis multiplex	14 (22.6)	Mild	15 (24.2)
Polyneuropathy	9 (14.5)	Moderate	2 (3.2)
Central nervous system vasculitis	6 (9.7)	Large	2 (3.2)
Lungs (excluding asthma)	42 (67.7)	Tamponade	4 (6.3)
Lung infiltrate	35 (56.4)	Pericarditis	7 (11.3)
Pleural effusion	13 (21.0)	Cardiomyopathy	44 (71.0)
Lung nodule	7 (11.3)	Myocarditis	23 (37.1)
Hemoptysis	4 (6.5)	Endomyocardial fibrosis	19 (30.6)
Tissue eosinophilia in lung biopsy	6 (9.7)	Intracardiac thrombus	14 (22.6)
Eosinophilia in bronchoalveolar lavage	2 (3.2)	Valvular regurgitation	8 (12.9)
Skin	18 (29.0)	Coronary arteritis	8 (12.9)
Rash	5 (8.1)	Gastrointestinal involvement	8 (12.9)
Purpura	8 (12.9)	Renal involvement	6 (9.7)
Skin nodule	3 (4.8)		
Phalange cyanosis, subungual petechiae	1 (1.6)		
Palmoplantar livedo	1 (1.6)		
Available skin biopsy positive for tissue eosinophilia	9 (14.5)		

Of the total cases, the LVEF was not reported in 9 patients. The mean LVEF of the remaining cases was 34.92±15.05% (range=9.0–74.0%). We classified the severity of LV dysfunction based on the LVEF. The LV function remained normal in only 10 (18.9%) cases. Mild (LVEF=41.0–51.0%), moderate (LVEF=30.0–40.0%), and severe (LVEF<30.0%) dysfunction was observed in 5 (9.4%), 18 (34.0%), and 20 (37.7%) cases, respectively.

Of the 43 cases with LV dysfunction at presentation, the follow-up LVEF was reported in 36 patients. In 26 (72.2%) cases, the LV function improved after treatment. The degree of improvement was variable and treatment had not always led to the normalization of the LV function. In 10 patients, the LV function did not improve. Of these cases, 1 patient suffered a malignant course of EGPA, which was complicated with intestinal perforation and alveolar hemorrhage. ^4^ This patient had the lowest LVEF reported of the whole study group and also the highest calculated FFS (=4). He, unfortunately, expired with sepsis as a result of aggressive immunosuppressive therapy. In another case, the LV function did not show improvement after treatment but the FFS was 1 and the patient eventually experienced a good clinical course after receiving combination therapy of prednisolone and mycophenolate mofetil. Symptoms of heart failure were controlled with medical therapy, and there was no need for advanced treatment.^61^ Of the remaining 8 cases with persistent LV dysfunction, all the patients underwent heart transplantation. Three of them finally expired with sudden death with evidence of EGPA relapse involving the transplanted heart in 2 of them. These expired patients underwent heart transplantation before the year 2010 and were reported in a case series by Groh et al.^26^ In a more recent case report, Rastogi et al.^62^ introduced a case of EGPA that underwent successful heart transplantation. Following a reduced dose of immunosuppressive therapy 18 months after the transplantation, relapse of EGPA became evident (peripheral eosinophilia and eosinophilic necrosis in endomyocardial biopsy). Through the intensification of the immunosuppression, this complication was managed successfully and the patient survived. This case showed improved outcomes for these patients in the modern era. 

Cardiogenic shock is an unusual presentation of EGPA. In our study population, only 7 (11.3%) patients presented with this life-threatening clinical picture.^13^^, ^^14^^, ^^19^^, ^^33^^, ^^34^^, ^^40^^, ^^60^ All of these cases recovered and survived after receiving appropriate treatment with/without mechanical circulatory support. The most common mechanism of cardiogenic shock was severe LV dysfunction due to eosinophilic myocarditis. Coronary vasculitis resulting in the diffuse spasm of the coronary arteries was reported in 2 cases.^33^^, ^^60^ In an interesting report by Kobayashi et al.,^14^ myocarditis resulted in severe LV hypertrophy and dynamic LV outflow tract obstruction, which also contributed to the shock state.

Of the patients presenting with chest pain, an initial diagnosis of acute coronary syndrome was made in 14 cases. Most of them subsequently underwent coronary angiography. Two of the patients with significant atherosclerotic coronary lesions were accordingly treated with revascularization (percutaneous coronary intervention in 1 and coronary bypass in the other) or medical therapy based on the suspected etiology. In a unique case reported by Cura et al.,^17^ refractory chest pain was successfully controlled with cardiac denervation in the patient with nonsignificant coronary lesions whose chest pain was probably due to coronary spasm. 

Intracardiac thrombus formation was a relatively common cardiac complication of EGPA in our study population which was discovered in 14 (22.6%) cases. It was seen with/without LV dysfunction. Intracardiac thrombus formation may be detected in any cardiac chamber, but the most common involved cavity was the LV. Francis et al.^44^ reported an interesting EGPA case with predominant right ventricular (RV) involvement. Myocarditis of the RV and an RV apical clot were detected by CMR. 

ECG findings were available for 54 patients. Of these, the ECG was abnormal in 37 (68.5%). The most common abnormality was ST-T changes, which were present in 32 (51.6%) cases. Various arrhythmias were detected in 12 (22.2%) patients including supraventricular/ventricular tachycardia and conduction abnormalities.

The overall prognosis of EGPA with cardiac involvement was good. In 52 (83.9%) of our patient population, optimal treatment eventually resulted in remission. Relapse after treatment was reported in 1 case, which was controlled after more intensive immunosuppressive therapy.^61^ Eight (12.9%) patients underwent heart transplantation after failure of medical treatment. Mortality was reported for 5 (8.1%) patients: 3 patients died after heart transplantation,^26^ 1 patient died with severe sepsis after intestinal perforation,^4^ and 1 patient due to ventricular fibrillation.^52^

## Discussion

We collected the clinical data of 62 patients suffering from EGPA with cardiac involvement from the literature and analyzed the frequency of various organ diseases in these patients. We also investigated the different laboratory and imaging methods for detecting cardiac involvement in patients with EGPA. Our study revealed subclinical cardiac involvement in only 17.7% of the patients. In the remaining symptomatic group, various clinical presentations could be expected. The most common cardiac presentations were heart failure (51.6%) and chest pain (32.3%). Only 6.3% of the patients presented with cardiac tamponade and 11.3% with cardiogenic shock. Palpitation was the only cardiac symptom in 2 (3.2%) patients. Intracardiac thrombus formation was a relatively common cardiac complication of EGPA in our study population (22.6%), and the most common involved cavity was the LV. ECG was abnormal in 68.5% of the patients with available ECG interpretation. The most common abnormality was ST-T changes (51.6%). Abnormal cardiac biomarkers were detected in 77.4% and abnormal echocardiography in 96.8% of the patients. CMR was done for 46.8% of the patients, and it was abnormal in all of them. Of the 16 patients who underwent endomyocardial biopsy, eosinophilic myocarditis was documented in 13 patients. We also evaluated the prevalence of extracardiac organ involvement in these patients. Asthma was present in 93.5% of the patients. The other commonly affected organs were the lung (67.7%), the ear-nose-throat (61.3%), the peripheral nervous system (37.1%), and the skin (29.0%). The less commonly involved organs were the GI tract (12.9%), the kidney (9.7%), and the CNS (9.7%).

EGPA is a systemic vasculitis affecting various organs including the heart. Based on the American College of Rheumatology 1990 criteria, EGPA is diagnosed if at least 4 of 6 criteria are present^64^: 1) asthma, 2) eosinophilia >10.0%, 3) mono- or polyneuropathy attributable to systemic vasculitis, 4) migratory or transitory pulmonary infiltrates detected radiographically (not including fixed infiltrates) and attributable to vasculitis, 5) paranasal sinus abnormality, and 6) extravascular eosinophil infiltration on biopsy. 

The reported sensitivity and specificity of these criteria for the diagnosis of EGPA are 85.0% and 99.7%, respectively.^64^

EGPA is classified as an ANCA-associated vasculitis. However, ANCA positivity occurs in only 40.0% of cases.^65^ Cardiac involvement can be detected in both ANCA-positive and ANCA-negative patients, although patients with more clinically overt heart involvement are mainly ANCA negative.^66^^, ^^67^

The pathogenesis of the disease is not clearly defined; however, it is often assumed to be an autoimmune disease due to the presence of ANCA.^65^ Among the ANCA-associated vasculitis, EGPA is more commonly associated with cardiac involvement.^66^ Cardiac involvement in patients with EGPA is a result of the direct activity of the disease affecting any part of the heart.^68^ Two main mechanisms explain this activity: vasculitis‑related ischemia and the eosinophilic infiltration of the myocardium.^9^

In a study by Sinico and Bottero,^69^ ANCA-positive patients were more likely to have necrotizing glomerulonephritis, mononeuritis, and purpura, whereas ANCA-negative cases were more likely to have cardiac and lung involvement. Furthermore, eosinophil tissue infiltration is more dominant in ANCA-negative patients.^23^

Dennert et al.^11^ in a study on 32 cases of EGPA, reported that subclinical cardiac involvement was more common than symptomatic cardiac involvement. They also reported symptomatic involvement in only one-fourth of the patients. This is in contrast to our study results as we found subclinical involvement in only 17.7% of our study population. This can be explained by the more active disease in our patient population. Dennert and colleagues stated that all their patients were in remission. Table 3 summarizes the possible presentations of patients suffering from EGPA with cardiac involvement. More than 1 feature may be evident in the same patient. 

**Table 3:** Cardiac involvement in EPGA

Site of involvement	Presentation/type of involvement
Pericardium	Pericardial effusion, tamponade, acute pericarditis, constrictive pericarditis
Myocardium	Acute myocarditis, cardiomyopathy, acute or chronic heart failure, cardiogenic shock, arrhythmia
Endocardium	Endomyocarditis, endomyocardial fibrosis, intracardiac thrombus formation
Valves	Valvular regurgitation (secondary to cardiomyopathy, leaflet or papillary muscle involvement)
Coronary arteries	Acute coronary syndrome (coronary vasculitis resulting in spasm or intracoronary thrombi)

EGPA, Eosinophilic granulomatosis with polyangiitis

Durel et al.,^70^ in a multicenter follow-up study of 101 patients with EGPA, aimed to assess the long-term outcome in these patients and concluded that the rate of cardiomyopathy did not differ according to ANCA status. The overall survival after a 6-year follow-up was 93.0%. An important finding was the protective effect of ear-nose-throat involvement for cardiac morbidity. In our study population, ear-nose-throat involvement was a common finding in that it was observed in 61.3% of the cases and the overall outcome was favorable, with remission achieved in 83.8% of the cases.

Even subclinical cardiac involvement has an adverse effect on the prognosis of patients with EGPA. Hazebroek et al.,^71^ in a prospective study of 50 patients with EGPA in sustained remission, aimed to investigate the prevalence and prognostic effect of cardiac involvement by applying an in-depth cardiac screening program consisting of ECG, 24-hour Holter, echocardiography, and CMR. They concluded that cardiac involvement was a strong predictor of mortality. ECG and echocardiography showed cardiac abnormalities in 62.0% of the patients. When CMR was also used, the prevalence of cardiac involvement reached 66.0%. In our study, we observed a much higher prevalence because we initially included EGPA cases with documented cardiac involvement; abnormal ECG and echocardiography were observed in 98.3% of our patients.

As was previously mentioned, any part of the heart can be involved by EGPA; therefore, the cardiac presentations of these patients are highly variable. It could be even a cause of sudden cardiac death in a previously healthy person.^72^ However, the usual presentations in many case reports were pericarditis, myocarditis, cardiomyopathy, heart failure, cardiogenic shock, myocardial infarction, and arrhythmia. Based on the studies of large EGPA cohorts, cardiomyopathy and pericardial effusion are the most common features of EGPA.^73^ The prevalence of pericardial involvement in previous studies is around 20.0%,^66^ but severe pericarditis or cardiac tamponade has rarely been reported.^16^ Comarmond et al.,^74^ in a long-term follow-up study of 383 patients suffering from EGPA, reported a 16.4% prevalence rate of cardiomyopathy in the total study group and 19.2% in the ANCA-negative subgroup. In our study, which was confined to patients suffering from EGPA with documented cardiac involvement, we observed cardiomyopathy and pericardial effusion in 71.0% and 37.1% of our cases, respectively. 

Other cardiac features such as coronary vasculitis and valvular dysfunction are less common and are restricted to some case reports. (We observed a 12.9% prevalence rate for each.) Studies that have performed imaging to evaluate the coronary arteries in patients with EGPA are scarce, and relevant data are, consequently, limited. Coronary vasculitis is often not diagnosed until postmortem. Coronary vasculitis was detected in 6 of 10 cases in the preliminary postmortem study by Churg and Strauss.^75^ Appropriate steroid/immunosuppressive therapy may induce complete angiographic regression of the disease.^76^

Cardiac involvement in EGPA may mimic acute coronary syndrome. ECG may reveal non-specific ST-T changes or, rarely, ST elevation due to coronary vasospasm or intracoronary thrombi.^18^ Coronary angiography can show stenotic lesions, coronary ectasia, or vasospasm. Coronary vasospasm has been considered the main cause of chest pain in patients with EGPA without significant coronary disease.^17^ Coronary ectasia is previously reported in many systemic inflammatory vasculitis, and it is associated with ectasia in other arterial beds such as the kidney and brain vasculature.^41^

The mean eosinophil percentage of our study population was high (44.29±21.10%). Higher eosinophilic counts are related to a higher prevalence of cardiac involvement. In a retrospective study by Neumann et al.^73^ analyzing 49 patients with EGPA, evidence of cardiac involvement was observed in 45.0% of the cases. Patients with cardiac involvement were compared with those without cardiac involvement, and a statistically significant difference was observed for the ANCA positivity and the eosinophil count. Patients with cardiac involvement were more ANCA negative and had higher eosinophil counts. 

The current study had some limitations. Some data were not available in all case reports such as BNP and NT-proBNP levels and we could not examine the relationship between these biomarkers and the severity of cardiac impairment. We only included cases with cardiac involvement. Thus, our study is a descriptive study reporting the prevalence of different cardiac lesions in these patients. What is missing, therefore, is a group of cases with EGPA without cardiac involvement so as to perform a statistical evaluation of the differences with the main study group. 

## Conclusion

The results of our study showed that any part of the heart could be involved by EGPA; therefore, cardiac involvement may present in a highly variable fashion. Furthermore, EGPA may also be subclinical. Regardless of the symptom status of patients with EGPA, an in-depth cardiac evaluation is warranted in all patients with a confirmed diagnosis of EGPA in order to detect cardiac involvement in its early phase and to start optimal treatment as soon as possible.

## Notes:

***This paper should be cited as: ***Pakbaz M, Pakbaz M. Cardiac Involvement in Eosinophilic Granulomatosis with Polyangiitis: A Meta-Analysis of 62 Case Reports. J Teh Univ Heart Ctr 2020;15(1):18-26.
